# Dental Hygiene

**Published:** 1885-07

**Authors:** G. L. Curtis


ARTICLE IV.

### DENTAL HYGIENE.

BY G. L. CURTIS, D. D. S.

**(Read at a Union Meeting of the Fifth and-Sixth District Dental Societies.)**

As is well known, a judiciously applied stopping will arrest decay and save a tooth for years of usefulness to its owner, but a course of treatment that will effectually prevent the first appearance of decay is as yet almost unknown, or at least unheeded, in our profession.

Constitution, health, nutrition, or habits of living, all bear so directly upon the teeth and their construction, that in this age of so-called civilization, with its artificial means of living, the dental organs have degenerated, until they seem almost beyond the reach of remedial agents. Local treatment, however, and constant care in daily life, will accomplish much.

Cleanliness, which is indispensable, and is certainly the most important remedy in preventing disintegration of the teeth, stands first, and the dentist cannot too earnestly impress upon the minds of his patients the importance of this great fact. Neglect this, and the advantages of good inheritance, natural strength, and the most skillful treatment, are all jeopardized.

The dentist should strongly urge the use of the brush after each meal, and before retiring, teaching the patient how to use it so as to get the upward and downward motion that enables the bristles to pass between the teeth and remove all deposits of food and calcareous deposits, and at the same time polish the approximating surfaces, instead of the lateral motion more commonly practiced, in which the labial surfaces only can be reached. Many would be benefited by having a proper brush selected for them, and should be instructed as to what dentifrice is not injurious.

The importance of the use of the quill pick, and of the floss silk, are second only to that of the brush, and for the purpose for which it is used the common quill pick is the best. The significance of all this, and the great importance of keeping the teeth clean, should be impressed upon the minds of children as well as of adults, and with all the earnestness possible. With ænemic and delicate persons it is often necessary to resort to general treatment. In prescribing medicine, which by direct contact would be injurious to the teeth, due care should be exercised, as we too often see the effect resulting from neglect in this particular, on the part of physicians. Alkaline washes are very beneficial in systems where the saliva is rendered acid in its reaction, and should be freely used. Our attention is often called to one or more teeth which yield readily to the secretions of the mouth, and become so sensitive that pain is produced whenever an attempt is made to brush them. In such cases lime water, bicarbonate of soda, or precipitated chalk, is useful. The latter should be placed around the teeth on retiring, and allowed to remain till morning.

To strike at the root of our subject, education is first essential, and every mother should be cognizant of the necessity of giving herself the proper care during gestation, and of taking the requisite amount of exercise and suitable food to develop the bone tissue of her child. This, too, is particularly important during the period of lactation, and the development of the permanent teeth.

In conclusion, we believe it is to the children and their teeth that we should direct our chief attention, and that in the carrying out of the best treatment known to us, together with what suggestions we may be able to get from those around us, lies our greatest success.—*Independent Practitioner.*

